# Thinking on Treating Echolalia in Aphasia: Recommendations and Caveats for Future Research Directions

**DOI:** 10.3389/fnhum.2017.00164

**Published:** 2017-04-03

**Authors:** Marcelo L. Berthier, María J. Torres-Prioris, Diana López-Barroso

**Affiliations:** ^1^Cognitive Neurology and Aphasia Unit and Cathedra ARPA of Aphasia, Centro de Investigaciones Médico-Sanitarias and Instituto de Investigación Biomédica de Málaga, University of MalagaMalaga, Spain; ^2^Area of Psychobiology, Faculty of Psychology, University of MalagaMalaga, Spain

**Keywords:** imitation, repetition, echolalia, aphasia therapy, memantine

## Are verbal imitation and repetition the same?

Imitation in the form of repeating speech sounds, accents, and words plays a foundational role in the normal acquisition and development of language (Meltzoff et al., 2009; Adank et al., 2013) eventually contributing to a life-long fine-tuning of communication skills (Tannen, 1987; Delvaux and Soquet, 2007). Imitation of prosodic and paralinguistic features may be intentional in certain contexts (e.g., mockery, impersonation, acting rehearsal). However, in general, imitation in healthy subjects is unintended as it involves automatic mimicry of non-essential components of the acoustic-phonetic information (speaking rate, prosody, accent) embedded in the heard message (Kappes et al., 2010)—the so-called *chameleon* effect. Therefore, it seems that verbal imitation is not the same as verbal repetition because in the latter, the auditory stimulus is intentionally repeated and the reproduced speech contains relevant phonological information, but the incidental acoustic features of the perceived stimulus are not invariably mimicked (Kappes et al., 2009, 2010).

## Echolalic repetition and its subtypes

Echolalia, the repetition of words and/or utterances spoken by another person (Wallesch, 1990), is frequently documented in individuals with autism spectrum disorders (Stiegler, 2015), neurodegenerative dementias (Da Cruz, 2010; Kertesz et al., 2010), post-stroke aphasia (Geschwind et al., 1968; Christman et al., 2004), and other neurologic and psychiatric disorders (Berthier et al., 2017a). However, there are no studies on the prevalence of echolalia in these conditions. This is intriguing as, for instance, echolalia is a usual accompanying feature of transcortical aphasias, which represent 4–20% of all aphasias (Berthier, 1999). Moreover, echolalia has occasionally been described during the recovery process of classical perisylvian aphasias (global, Wernicke, conduction, Broca; Brown, 1975; Hadano et al., 1998; López-Barroso et al., 2017). This implies that a more in depth assessment would inflate the prevalence rates.

Echolalia is a heterogeneous symptom of aphasia and several subtypes have been described (Wallesch, 1990; Berthier, 1999). More than one type of echolalia can coexist in the same patient (Brown, 1975; Hadano et al., 1998) and changes from one form to another (i.e., from *complete* to *partial*) during aphasia evolution is common. The most severe types of echolalia occur in aphasias with preserved repetition abilities (transcortical aphasias; Berthier et al., 2017a). Two of them, *ambient echolalia*^1^ and *echoing approval*^2^ are chiefly characterized by the production of echoes of comments and questions not directed to the patient but to other people. Such disinhibition, elicited by merely hearing speech in the environment, results from diffuse brain injury (Geschwind et al., 1968) or extensive unilateral or bilateral lesions in medial frontal and anterior cingulate cortices and subcortical structures (Ghika et al., 1996; Suzuki et al., 2009, 2012). In both forms, deficient inhibition of repetition conceivably results from altered control of shared representations (misunderstanding the intentions of others; Frith and Frith, 2006; Brass et al., 2009; Besnard et al., 2011) and evaluation of outcomes (e.g., impaired reflection on one's own performance; Passingham et al., 2010; Berthier et al., 2017a). At variance with the abovementioned types of verbal echoing, another severe form named *automatic echolalia*^3^ is provoked when patients are directly addressed, and not when comments and questions are directed to other people. This suggests better control of shared representations (self-other distinctions). Awareness about the irrepressible echoing may or may not be preserved, but note that these cognitive domains have not been formally investigated so far. Automatic echolalia in aphasia usually occurs after lesions in the left hemisphere placed outside the perisylvian language area (PLA; the *isolation of the speech area* hypothesis) responsible for verbal repetition. This hypothesis, early championed by Goldstein (1948) and Geschwind (Geschwind et al., 1968), maintains that echolalic repetition in aphasia occurs because the left PLA is anatomically intact, but out-of-control by virtue of being disconnected from close and distant eloquent cortical regions (SMA, temporo-parietal cortex) underlying language production and comprehension. Nevertheless, other mechanisms (*right hemisphere or bilateral* hypotheses) underlying echolalic repetition have been proposed (Niessl von Mayendorf, 1911; Brown, 1975). Modern studies provided evidence that in such cases the left PLA area may be dysfunctional (Berthier et al., 1997) with limited competence to generate verbal repetition and echolalia (López-Barroso et al., 2017). In this situation, verbal echoing most likely depends on the vicarious activity of the right hemisphere (López-Barroso et al., 2017). In support, studies in healthy volunteers using functional neuroimaging (Saur et al., 2008) and transient virtual lesions over the left inferior frontal gyrus (Hartwigsen et al., 2013) revealed bilateral temporofrontal participation during repetition of words and increased activity in the contralateral homologous area during repetition of nonwords, respectively. Another piece of evidence that supports the right hemisphere hypothesis is the case of formerly globally aphasic patients with large left PLA lesions, who develop automatic echolalia years after aphasia onset through gradual remodeling of right hemisphere networks (Pulvermüller and Schönle, 1993; Berthier et al., 1997). In keeping with these findings, a right intracarotid amobarbital injection (Wada test) suppressed automatic echolalia in a case of transcortical sensory aphasia and left hemisphere damage (Case 1 in Berthier et al., 1991). In this regard, what requires elucidation is why only a small proportion of patients with severe aphasia after left extensive hemisphere damage develop echolalia. A tentative explanation is that individual differences in the status of repetition, and hence on the possibility of developing verbal echoing in aphasia, may depend on both the premorbid structure of gray matter (Xing et al., 2016) and variability of right white matter tracts (Catani et al., 2007; Berthier et al., 2012; Forkel et al., 2014).

Two less severe forms of verbal echoing have been designated as *mitigated echolalia*^4^ (Pick, 1924; Lebrun et al., 1971) and *effortful echolalia*^5^ (Hadano et al., 1998). Information on these variants is scarce, but one distinctive element is that they are also observed in aphasias with impaired verbal repetition (conduction aphasia, Wernicke's aphasia, Broca's aphasia). The responsible lesions involve the left temporo-parietal cortex in mitigated echolalia and large portions of the left PLA in effortful echolalia. It is apparent that mitigated echolalia entails better control over the echoed material than in the more severe forms as reflected by the introduction of changes in the reproduced emissions compared to the verbatim repetition that accompanies, for example, automatic echolalia. Modifications in wording or intonation on the echoed emissions may have different purposes such as recapitulate meaning, regain attention, take time to plan a response, reinforcement of an idea, contradict, complement the just received message, or empathize with the interlocutor. However, despite the general consensus that the production of echoes of words and phrase fragments is aimed to resolve impaired access to word meaning during auditory comprehension, deficits in auditory-verbal short-term memory and incompetent inhibitory control have also been described (Berthier et al., 2017b). Thus, it seems that mitigated echolalia is not always in the service of improving auditory comprehension.

Little information also exists on the other type, effortful echolalia. It is essentially a form of mitigated echolalia, yet the production of echoes is laborious and limited to short phrase fragments produced with dysarthria and distorted prosody (Hadano et al., 1998). At present, there is no information on whether effortful echolalia helps the very limited communication ability or whether it merely represents a disinhibition symptom. In the few cases reported up to now, effortful echolalia results from simultaneous involvement of the left supplementary motor area and left PLA (e.g., Broca's area, anterior insula; Hadano et al., 1998). While verbal echoes after damage to the left supplementary motor area in other forms of echolalia are produced with fluent and well-articulated speech, the laborious production in effortful echolalia reflects the additional involvement of the left anterior PLA. Awareness on the irrepressible character of echoes seems to be variable and needs further evaluation.

## Neural mechanisms

Nowadays the neural mechanisms supporting verbal imitation/repetition (Mashal et al., 2012) and their inhibition in inappropriate situations (Bien et al., 2009; Aron et al., 2014) are relatively well-known. Progress in the study of network models for action observation and imitation of speech in healthy subjects suggest that action understanding, imitation, and verbal learning requires an orchestrated coordination of different brain region in which the mirror neuron system (MNS) and the white matter tracts linking its different nodes are involved (Kohler et al., 2002; Arbib, 2010; but see criticisms to the role of MNS in Hickok, 2009; Mikulan et al., 2014). The audio-visual MNS is located in ventrolateral prefrontal cortex, superior temporal gyrus, and inferior parietal lobule overlapping with the dorsal speech-processing stream, and these areas are linked via the arcuate fasciculus (Arbib, 2010; Corballis, 2010). The audio-visual MNS represents a mechanism for integrating perception and action, which fits well with preferential role of the dorsal stream, involved in automatic non-semantic translation from the sensory to the motor code (i.e., auditory-motor integration), required for voluntary verbal repetition, short-term memory, and verbal learning (Hickok and Poeppel, 2007; Rodríguez-Fornells et al., 2009; López-Barroso et al., 2013, 2015). This intricate neural system operates under the supervision of a bilateral executive-control network (premotor, posterior parietal and frontal-parietal opercular cortices, right inferior frontal, and superior temporal cortices, and basal ganglia), which acts as a “brake” supressing inappropriate, automatic overt repetition (echolalia; Aron et al., 2014; Bien et al., 2009). When brain pathology abolishes the regulatory function of these areas in the left hemisphere of patients with aphasia, their verbal repetition is out of control and echolalia ensues by virtue of automatic activation of action-perception circuits including the audio-visual MNS (Berthier et al., 2006, 2017a). The mechanism is possibly more complex in bilingual and polyglot patients with aphasia who repeat a just heard verbal material but in a different language [examiner: “*What time is it?”*; patient response: “*Quelle heure est-il?”* (Veyrac, 1931; see review in García, 2015)]. Nevertheless, imitation of paralinguistic features (prosody) is not always possible and repeated words and sentences sound flat and devoid of emotional coloring (Speedie et al., 1984; Berthier et al., 1996; Kappes et al., 2009), thus suggesting that repetition and imitation are dissociable. Alternatively, less severe forms of echolalia are produced in a voluntary manner and thus are not directly associated to a grossly abnormal functioning of this regulatory system.

## Broadening the scope of testing for echolalia

The notion that impairments in non-language cognitive domains and behavior influence the clinical presentation and evolution of aphasia is gaining credence amongst aphasiologists (Kauhanen et al., 2000; Fucetola et al., 2006; van de Sandt-Koenderman et al., 2008; Lambon Ralph et al., 2010; El Hachioui et al., 2014). Since the same argument probably holds for echolalia in aphasia, we emphasize the strong necessity to explore the relationship between verbal echoing, concurrent deficits in language and high-level cognitive non-language processes, and the neural mechanisms underpinning these domains in aphasia. Our proposal is that the analysis of this interaction would provide hints for devising neurorehabilitation strategies tailored to each patient needs, trying to be consistent with the current function of echolalia and its potential instrumental role in relation to functional communication.

**Figure 1:**
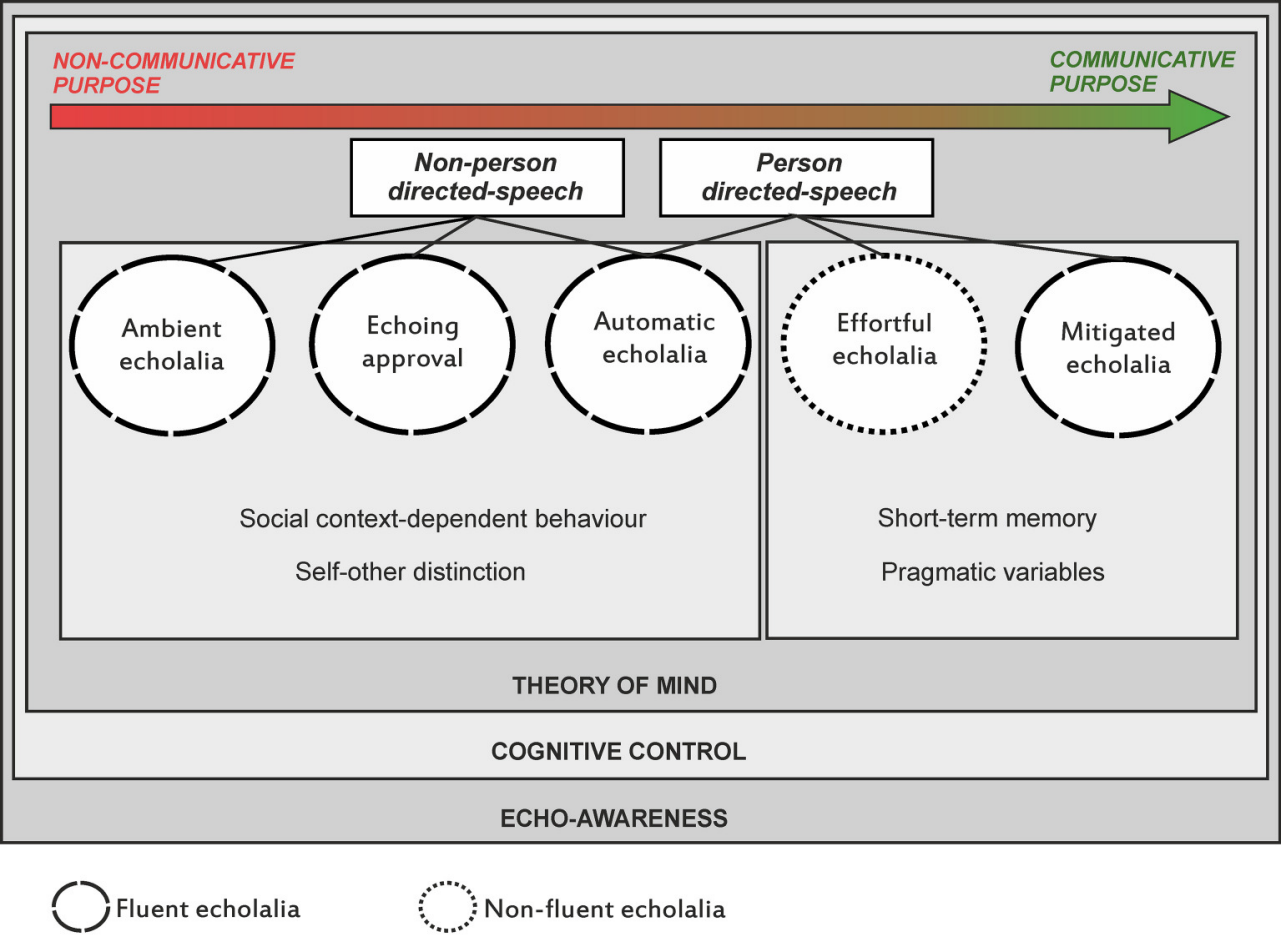
**A scheme depicting the continuum of severity of echolalia types ranging from severe (non-communicative purpose) to mild (communicative purpose) forms together with the identification of precipitants stimuli (environmental or personal)**. The Figure also summarizes a proposal of non-linguistic cognitive functions (e.g., self-other distinction, pragmatic variables) that could be evaluated in each type to help identify the diverse deficits underlying echolalia and the development of treatment strategies. Evaluation is also required in potential deficits affecting theory of mind, cognitive control, and echo-awareness, which are probably more generally involved in all forms. A distinction between types of echolalia produced with fluent and effortless pattern and another emitted with non-fluent and laborious speech is also shown.

Through the years, it has been advocated that deficits underpinning echolalia are related to breakdown of various domains including inhibitory control, mentalizing (theory of mind), decision making, awareness, auditory comprehension, auditory-visual feedback, and auditory-verbal short-term memory (see Berthier et al., 2017a). Nevertheless, the relative contribution of each deficit to the different types of echolalia remains unexplored. The Figure 1 summarizes the existing types of echolalia together with a proposal of the non-linguistic cognitive and behavioral functions that could be involved in this complex symptom and that we suggest to explore in each type.

## Do all types of echolalia require treatment?

One key issue that needs elucidation is whether all types of echolalia associated with aphasia require treatment. Moreover, in the case that one advocates a therapeutic intervention for echolalia, the question is what to do with it, *inhibit* or *reshaping*? The answer of this largely unexplored issue is far for being contested and is probably more complex than it may appear. Echolalia is a symptom that appears in a wider clinical context, very often presented together with comprehension, fluency or short-term memory deficits, amongst other non-language cognitive deficits (Figure 1). In addition, the degree of control over the repeated material is subjected to changes on a severity continuum from an uncontrollable automatic echoing to a more indolent voluntary repetition. This would imply that the faulty inhibition of impulsive echolalia seen in some cases contrasts sharply with the voluntariness to repeat verbal material seen in other cases, mostly to improve auditory comprehension, in which the content of verbal echoes is not always a verbatim reproduction of what has been heard. This poses the question of how and when echoes have unfavorable or beneficial effects on aphasia. The extant evidence suggests that there are not determinant answers that will be suitable for all cases. The whole clinical profile of each patient should be considered. The more severe variants of echolalia are often highly disruptive and need to be directly targeted in the rehabilitation process. Nevertheless, the evidence suggests that in cases wherein automatic echolalia in non-fluent transcortical aphasias is the only available channel for verbal production, efforts to redirect and incorporate echoes in the service of speech production and comprehension using therapies tailored to modulate the activity of action-perception links (e.g., Constraint-Induced Aphasia Therapy—CIAT) are useful (Pulvermüller and Schönle, 1993; Kurland et al., 2012). The picture is not as clear for the less severe types (mitigated and effortful). Even when, in many cases, echolalia may be functional and, for example, facilitate comprehension, the incessant repetition of auditory stimuli may interfere with functional communication and make evaluations excessively long (Berthier et al., 2017b). In a recent single case study of a patient with residual Wernicke's aphasia, mitigated echolalia was significantly reduced using CIAT (supplemented with verbal instructions made by the therapist to attenuate imitative tendencies) and a cognitive-enhancing drug (memantine; Berthier et al., 2017b).

The recent identification of a neural network for action observation and imitation of speech (Mashal et al., 2012) provided a theoretical framework for developing new model-based therapies for aphasia, namely *IMITATE* (Intensive Mouth Imitation and Talking for Aphasia Therapeutic Effects; Lee et al., 2010; Sarasso et al., 2014; Duncan and Small, 2016) and *SPEECH ENTRAINMENT* (Fridriksson et al., 2012). These interventions aim to improve speech production through action observation and audio-visual feedback via verbal repetition-imitation, which recruits the dorsal and ventral streams in both cerebral hemispheres (Lee et al., 2010; Fridriksson et al., 2012; Sarasso et al., 2014; Chen et al., 2015). Preliminary evidence indicates that this treatment approach facilitate recovery of speech production in different types of aphasia, including in cases of non-fluent transcortical aphasias, which are usually associated with echolalia, by inducing plastic changes in both cerebral hemispheres (Sarasso et al., 2014; Chen et al., 2015). However, these preliminary studies did not clarify if treated patients actually had echolalia. This is important because non-invasive overstimulation of the MNS in the left inferior frontal gyrus facilitates verbal repetition (Restle et al., 2012) and stimulation of fronto-median areas, which exerts a top-down inhibitory control over the MNS, induces echophenomena (Finis et al., 2013). Therefore, it remains to be determined whether therapies like *IMITATE* and *SPEECH ENTRAINMENT* tailored to strengthen the activity of the MNS are applicable to aphasic patients with echolalia. In any case, more studies are needed to determine whether reshaping the activity of the observation-imitation networks may redirect echolalia to the service of spontaneous speech in cases of non-fluent aphasias.

## Conclusions

In this opinion article, we have analyzed the current state-of-the-art of echolalia in aphasia. We aimed to enlighten some recommendations to gain insight on diagnosis, neural mechanisms, and treatment of echolalia as well as to call attention on caveats that merit attention and analysis. Studies of prevalence are warranted because echolalia is very frequent in degenerative dementias coursing with aphasia (Alzheimer's disease, semantic dementia) and because neuropharmacological interventions can attenuate these symptoms in patients with Alzheimer's disease (Asp et al., 2006). Understanding the relationship of the different types of echolalia with aphasia is paramount to design adequate methodology for assessment and treatment strategies. At present, the analyzed data suggest that echolalia interfering with functional communication should be inhibited, whereas when echolalia is the only available verbal channel in aphasic cases with non-fluent speech it could be redirected to gradually convert such disinhibited speech into a meaningful communicative function.

## Author contributions

All authors listed, have made substantial, direct, and intellectual contribution to the work, and approved it for publication. MB, MT, and DL drafted the article and revised it critically for important intellectual content.

### Conflict of interest statement

The authors declare that the research was conducted in the absence of any commercial or financial relationships that could be construed as a potential conflict of interest.

## Footnotes

^1^The term ambient echolalia (Fisher, 1988) is applied when patients repeat words and sentences coming from unrelated conversations around them even when people are talking in a nearby room (Suzuki et al., 2012).

^2^The term echoing approval is used for those patients who imitate the affirmative or negative syntactical construction of questions or the intonation patterns even when questions are directed to other persons (environmental-dependency syndrome; Ghika et al., 1996).

^3^Automatic echolalia refers to the production of echoes in an impulsive, “parrot-like” manner. Patients do not appear inhibited by any type of verbal information including non-words or foreign languages.

^4^Mitigated echolalia refers to any language change in the echoed emission for communicative purposes (Pick, 1924), but recent data suggest that this is not always the case (Berthier et al., 2017b).

^5^Effortful echolalia denotes the articulatory struggling, distorted prosody, and increased effort observed in the echoes (Hadano et al., 1998).
